# Next Generation Sequencing of Fecal DNA Reveals the Dietary Diversity of the Widespread Insectivorous Predator Daubenton’s Bat (*Myotis daubentonii*) in Southwestern Finland

**DOI:** 10.1371/journal.pone.0082168

**Published:** 2013-11-27

**Authors:** Eero J. Vesterinen, Thomas Lilley, Veronika N. Laine, Niklas Wahlberg

**Affiliations:** Department of Biology, University of Turku, Turku, Finland; University of Milan-Bicocca, Italy

## Abstract

Understanding predator-prey dynamics is a fundamental task in the evaluation of the adaptive capacities of species. However, direct observations or morphological identification of fecal remains do not offer an effective way to study the dietary ecology of elusive species, such as nocturnal insectivorous bats. However, recent advances in molecular techniques have opened a new method for identifying prey species from fecal samples. In this study, we amplified species-specific mitochondrial COI fragments from fecal DNA extractions from 34 individual Daubenton’s bats (*Myotis daubentonii*) collected between 2008 and 2010 from southwestern Finland. Altogether, 128 different species of prey were identified based on a comprehensive local DNA reference library. In our study area, Daubenton’s bats feed most frequently on insects of the orders Diptera (found in the diet of 94% individuals), Trichoptera (69%) and Lepidoptera (63%). The most frequent dipteran family in the diet was Chironomidae, which was found in 31 of 34 individuals. Most common prey species were chironomids *Microtendipes pedellus* (found in 50% of bats), *Glyptotendipes cauliginellus* (44%), and *Procladius ferrugineus* (41%). For the first time, an accurate species level list of the diet of the insectivorous Daubenton’s bat (*Myotis daubentonii*) in Finland is presented. We report a generally applicable method for describing the arthropod diet of vertebrate predators. We compare public databases to a national database to highlight the importance of a local reference database.

## Introduction

Molecular analysis of diet from fecal samples offers a non-invasive tool to study the biology and ecology of a variety animals [1–6]. In bat ecology the basic questions are still largely unanswered. It is known that all of the bats in the most species rich bat family, the vesper bats (Vespertilionidae), feed on insects and other arthropods [7], but species level data of the diet is incomplete or lacking detail [8–14]. Thus, the first step in dietary analysis of bats is to compile an accurate prey species list [15]. Due to their nocturnal and aerial lifestyle, direct observations of relations between bats and insects are especially difficult in the natural environment, although there have been attempts to study this [16]. Also, insectivorous bats chew their food effectively and morphological species-level identification of prey from the fecal material of predators is difficult to achieve (but see 17). Consequently, due to the aforementioned obstacles, almost all published data on the diet of insectivorous bats rarely provide identifications beyond the level of order.

Recently, advances in DNA sequencing technologies have enabled molecular approaches to be applied to fecal samples in order to unveil inter-trophic relationships in a variety of species including sea lions [6], seals [1], sheep [18], mammals, birds, invertebrates [19] and bats [3,9]. Because of their high taxonomic resolution, up to species level, molecular diet studies have taken advantage of DNA barcoding regions [20] which also enable the use of DNA barcode libraries such as the Barcode of Life Database (BOLD) [21]. However, DNA obtained from fecal samples is highly degraded, which severely affects the amplification success of larger prey DNA segments [22]. The earlier feeding trials show that by using group-specific primers it is possible to amplify and identify almost all of the prey species from fecal samples [3,6]. For this reason, and in order to exclusively target prey DNA from fecal samples arthropod-specific primers amplifying a highly variable 157 base pair long segment of the DNA barcode have been developed [3].

Here we employ molecular, next generation sequencing techniques to describe the diet of 34 Daubenton’s bat individuals using the Ion Torrent PGM [23,24]. The Daubenton’s bat (*Myotis daubentonii* Kuhl 1817) is a small Eurasian bat, which has its northernmost European distribution in Finland. The majority of the animals hunt over water or in the vicinity of water, but exceptions have been observed in forests, parks or orchards. Daubenton’s bats are regarded mainly as trawling bats, meaning that insects are caught directly from the water surface. Their diet is thought to consist mostly of newly-hatched adult chironomids, but also other dipterans, caddisflies and arthropods are seasonally captured [7,10,13,25–32].

One necessary pre-requisite for accurate species identification is the availability of a comprehensive DNA barcode library of potential prey, which has been missing from previous studies so far [33]. Earlier studies have mainly relied on public databases such as GenBank [34] or BOLD. Here we take advantage of a comprehensive database on Finnish insect DNA barcodes (FinBOL; www.finbol.org), which includes our own DNA barcodes specifically generated for potential prey species of the bats, to investigate the diet of Daubenton’s bats in southwestern Finland. In summary, we attempt to answer these study questions:

1 Are the modern sequencing technologies (in the form of Ion Torrent PGM) suitable for sequencing fecal DNA for dietary studies?
2 What is the advantage of a comprehensive local DNA barcode library when using a molecular approach to dietary research?
3 What prey species does the Daubenton’s bat consume at the northern edge of its distribution?

## Materials and Methods

### Ethics Statement

The Daubenton's bats were sampled with active sampling licenses from the Finnish National Animal Experiment Board (ESLH-YM-2007-01055) and Centre for Economic Development, Transport and the Environment (LOS-2007-L-182-254). Care was taken to handle and trap bats according to ethical guidelines presented by the National Animal Experiment Board.

### The study area and its characteristics

**Figure 1:**
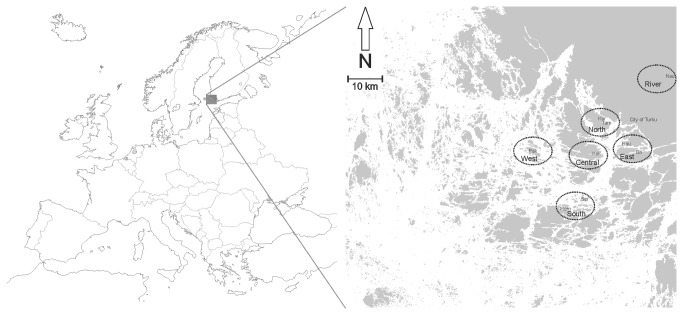
The study area, sampling sites, and regions. River (Nautelankoski rapid), North (Korjaustelakka and Tamminiemi), West (Pakinainen), Central (Kukonpää), East (Rauhala and Brinkhall), and South (Seili and Hemsundet). Land is shaded grey and water is white.

The sampling took place on the mainland and islands in the northern part of the Archipelago Sea, situated in southwestern Finland (Figure 1). The area has a large river outlet of fresh water and the salinity is very low (typically less than 5 psu) [35], which affects the aquatic as well as terrestrial species composition of the area, including insects with aquatic life stages. Due to the nutrient runoff from rivers, the Archipelago Sea is eutrophic [36]. The coastline in the northern part of the Archipelago Sea is covered by a wide (up to approx. 30 m) bed of Common reed (*Phragmites australis* (Cav.) Trin. ex Steud). Uncluttered areas in the vicinity of the reed beds are primary feeding areas for Daubenton’s bats in the area.

### Bat trapping and fecal sampling

Bats were caught with a combination of mist nets and harp trap. A Sussex Autobat acoustic lure, which plays species-specific ultrasound social calls, was placed in the center of harp trap to attract the bats [37]. This multi-trap combination was placed across the flying corridor of bats commuting between roosts and foraging areas.

All fecal sampling was conducted in the field. Upon catching, bats were kept in clean cloth bags until handling for identification and sampling. Caught bats were identified to species and sex and banded. Age was determined from bone structures, especially from the joint of phalanges. The bones of juveniles are not fully ossified. With the onset of flight, the phalanges are almost completely ossified, but growth plates near the joints are apparent as a light cartilaginous gap. Fecal pellets were collected either from laundered, individual handling bags or directly from the bat while handling. In the first case (bags) the fecal droppings were collected using disposable tweezers immediately when the bat was removed from the bag. In the second case (direct collecting) the fecal droppings were collected immediately upon excretion from the surroundings of anus using disposable tweezers. The pellets were then stored in 1.5 ml, 94 % ethanol-filled Eppendorf tubes, which were individually labeled and subsequently stored at +4. See Table 1 for detailed information of the sampled bat individuals.

**Table 1:** Bat individuals sampled for the study.

**Band nr**	**sex**	age*	**Sampling Date**	**Site**	**Accession**
868	♂	J	June 3, 2010	Tamminiemi	SRR949284
878	♂	J	July 31, 2008	Hemsundet	SRR947872
889	♀	A	June 3, 2010	Tamminiemi	SRR949301
1086	♀	A	June 3, 2010	Tamminiemi	SRR949312
1373	♂	J	May 26, 2009	Tamminiemi	SRR947874
1673	♂	J	July 27, 2009	Brinkhall	SRR949279
1706	♀	J	August 14, 2009	Pakinainen	SRR949282
1778	♀	J	July 29, 2009	Korjaustelakka	SRR949280
1790	♀	J	August 13, 2009	Brinkhall	SRR949281
1829	♂	A	August 26, 2010	Rauhala	SRR949292
1851	♂	J	July 16, 2009	Tamminiemi	SRR947875
1973	♂	A	July 25, 2010	Korjaustelakka	SRR949293
2015	♂	A	May 26, 2010	Tamminiemi	SRR949283
2026	♀	A	July 16, 2010	Korjaustelakka	SRR949294
2038	♀	A	July 20, 2010	Tamminiemi	SRR949295
2045	♀	A	July 23, 2010	Brinkhall	SRR949286
2053	♀	J	July 27, 2010	Korjaustelakka	SRR949296
2062	♂	A	July 28, 2010	Hemsundet	SRR949297
2066	♂	A	July 28, 2010	Hemsundet	SRR949287
2070	♂	A	August 1, 2010	Kukonpaa	SRR949288
2079	♂	A	August 3, 2010	Hemsundet	SRR949298
2080	♂	J	August 3, 2010	Hemsundet	SRR949310
2082	♂	J	August 3, 2010	Hemsundet	SRR949311
2099	♂	A	August 5, 2010	Nautelankoski	SRR949289
2407	♂	A	August 10, 2010	Seili	SRR949306
2409	♀	A	August 10, 2010	Seili	SRR949307
2411	♂	A	August 10, 2010	Seili	SRR949308
2413	♀	J	August 11, 2010	Seili	SRR949309
2434	♂	A	August 18, 2010	Rauhala	SRR949299
2435	♂	A	August 18, 2010	Rauhala	SRR949302
2437	♀	A	August 19, 2010	Rauhala	SRR949303
2438	♂	J	August 19, 2010	Rauhala	SRR949300
2439	♂	A	August 19, 2010	Rauhala	SRR949304
2479	♂	A	September 6, 2010	Rauhala	SRR949290

* J = juvenile, A = adult

Individual band number, sex, age, time, site of sampling and SRA accession code for the raw reads originating from faeces of each bat individual.

### DNA extraction and PCR setup

Altogether, we sampled 37 fecal pellets from 34 bat individuals for fecal DNA extractions (Table S1). We took one fecal pellet at a time from each individual to extract DNA. In order to avoid contamination, fecal pellets were processed in the Herbarium laboratory at the University of Turku, where only plant specimens have been processed so far. Total fecal DNA was extracted using QIAamp DNA Stool Mini Kit (Qiagen, catalog number 51504). Negative control extractions containing all the chemicals but no fecal pellets were performed alongside each batch of extractions to monitor for contamination of the extraction chemicals. The fecal DNA concentrations ranged from less than 1 to over 20 nanograms per microliter. The PCR reactions were also prepared in the insect-DNA-free laboratory and thermocycling was later performed in the TEGlab facilities (Laboratory of Genetics, University of Turku (for a review of methods, see 4). The following PCR conditions were applied to our DNA templates: 1x KAPA HiFi Hotstart ReadyMix (Kapa Biosystems, catalog number KK2602), 10 µM each dNTP, 8 % (final concentration) BSA, 0.5 µM each primer (ZBJ1c and ZBJ2c after [3]), and 1–2 µL fecal DNA extraction. PCR grade water was added up to 25 µL reaction volume. Negative control reactions were performed alongside each batch of PCR to monitor for contamination of the chemicals used. The thermal profile included a 5 min initial denaturation at 95 °C followed by 40 cycles of a 30 s denaturation at 95 °C, a 30 s annealing at 50 °C, and a 30 s elongation at 72 °C. Final elongation was conducted at 72 °C for 5 minutes. The PCR success was visually inspected under UV light using a 2 % agarose gel stained with Midori Green Advanced (Nippon Genetics Europe, catalog number MG04). The successful reactions were purified using NucleoMag 96 PCR Kit (Macherey-Nagel, catalog number 744100.1). The final elution volume was 25 µL.

### Ion Torrent PGM library preparation

We employed a time and cost effect method to pool individually tagged DNA samples into two sequencing libraries [38]. The first sequencing library included 15 individuals, and the second 23 individuals. Three individuals (band numbers 2015, 2026, and 2038) were included in both libraries. Libraries were prepared according to [38] with the following modifications to their protocol to make it more suitable for Ion Torrent PGM (steps as in [38]):

Step 1: The adapter mix was prepared using multiplex identifier (MID) tagged adapters. Adapters were designed in order to include an individual MID and to match Ion Torrent specific priming sites. The molar concentration of the adapter mix was 20 µM for each adapter. We used a 200 bp long PCR product as positive control DNA template through steps 1 to 26.

Step 4: We performed the blunt-end reaction with half of the volume, i.e., we added 10 µL of blunt-end master mix to 25 µL of sample. The next purification using SPRI beads was adjusted accordingly to ensure a template:bead ratio of 1:1.8. In the post-ligation purification steps template:bead ratio was 1:1.

Step 20: To verify adapter ligation success, the SPRI-purified MID-tagged positive control library and a subset of MID-tagged library aliquots were separated side by side with non-MID-tagged templates on 2% agarose gel stained with Midori Green Advanced electrophoresis (45 min, 95 V) and visualized using UV light.

Step 26: For a subset of samples, we measured DNA concentration and fragment size distribution using Bioanalyzer (Agilent Technologies, Inc.) and DNA concentration using Qubit Fluorometer (Life Technologies Corporation). The results from Qubit were consistent with Bioanalyzer results. We omitted Bioanalyzer run for each individual samples and quantified the libraries using the Qubit Fluorometer. Individual amplicon libraries were subsequently mixed in equimolar ratios.

The stock library was re-amplified in two separate reactions with following setup: 5 µL pooled library stock was added to a master mix consisting of 5 U of Herculase II polymerase (Agilent technologies, catalog number 600677), 1x Herculase II reaction buffer, 25 mM each dNTP, 10 µM each primer, and added PCR grade water up to 50 µL. We designed the re-amplification primers based on the sequence of the IonTorrent specific priming sites and thus generated millions of MID-tagged copies including binding sites necessary for subsequent sequencing utilizing IonTorrent technology. The thermocycling profile included a 30 s denaturation at 98 °C followed by 15 cycles consisting of a 20 s denaturation at 98 °C, a 30 s annealing at 64 °C, and a 30 s elongation at 72 °C. Final elongation was conducted at 72 °C for 5 minutes.

To clean the re-amplified library, size-selection was done by separating the entire library using 2% Size-Select Agarose E-Gel and E-Gel Electrophoresis System (Life Technologies, catalog numbers G6610-02 and G6500) following the manufacturer’s instructions. The DNA concentration of amplified library pool stock was again measured with Qubit Fluorometer. The library pool stock was then diluted to a final concentration of 26 pM. For template preparation, an 18 µL aliquot of the library dilution (approximately 2.8 x 10^8^ molecules) was transferred into the sequencing reaction set up.

Emulsion PCR and Ion Torrent PGM Sequencing were carried out on two 314 chips (Life Sciences, catalog number 4462923) according to the manufacture’s protocol (Publication Part Number: 4471974 Rev. C).

### Processing of Ion Torrent PGM reads

The resulting reads were binned and renamed by MID, i.e., the original individual samples using the software Geneious Pro 6.1 [39]. Then the original arthropod-specific primers (ZBJ-1c and ZBJ-2c; [3]) were trimmed off and all reads were trimmed for poor quality parts using 0.05 error probability limit and then the reads shorter than 100 bp were excluded using Geneious Pro 6.1. Thus, only reads containing both the Ion Torrent adapter with a MID and the original primer were passed on for further analysis. Subsequent analyses were carried out using super computers at the IT Center for Science (Espoo, Finland, www.csc.fi). Sequences were assigned to species using BLASTN 2.2.25+ algorithm [40,41] against two databases, built the following way: DB-1) a public database consisting of all the arthropod COI Barcode of Life Data systems [42] and Genbank [34], and DB-2) all the sequences as in database one plus sequences from National Finnish Barcode of Life (FinBOL) campaign and sequences previously generated by ourselves [43]. We blasted all individuals separately against DB-2 and we also blasted all the query sequences against both databases using super-computing servers at Finnish Grid Infrastructure (FGI, http://www.csc.fi/english/collaboration/projects/fgi). 

### Analyzing the BLAST results

The BLAST output was imported into the program MEGAN [44] to get an overview of the results. We used default parameters, except for following settings: Min support=1, Min score=100, Min complexity=0. We utilized a built-in comparison tool in MEGAN for database comparison. As recommended by [8,9], we discarded all hits below 98% identity. We also discarded hits with e-value over 1e-20. We further examined the hits one by one to see if there were reads matching more than one species in the database. In these cases we followed these steps:

1 Select the hit with the longest alignment length,
2 Select a match to an adult specimen over juvenile,
3 Check the database entry for errors and omit wrong entry

Following these simple steps we were able to reliably select species identity for 23253 reads. We calculated prey species accumulation curve [45] based on samples (= individual bat fecal pellets) to analyze the total dietary species richness using program EstimateS (version 9) using 1000 runs for sample order randomization ([46], see also [47] and references there on). The expected species curve confidence limits where calculated by rarefaction after [48]. Non-parametric Chao 1 (using classic formula setting) [49] and ICE [50,51] richness estimators were calculated and plotted against individual samples to see the behavior of traditional estimators for bats as diversity sampling units. The effective number of species (true diversity of order 1) was calculated after [52]. The dominance-diversity relationship of dietary species was analyzed by plotting species occurrences per species against species rank (from most abundant to least abundant). The differences in species diversity between regions could not be statistically analyzed due to small sample size and biased sampling. To visually demonstrate the prey species assemblages we drew a species cloud based on the identification and frequency of all the prey species at each region.

## Results

### Overview of Ion Torrent PGM data

Two sequencing runs produced 12.1 Mb of raw data of which 8.67 Mb showed a quality of Q20 or higher (see Supporting information S1 and S2 for details). The raw reads are deposited in the Sequence Read Archive (SRA). The SRA accession codes for each individual are listed in Table 1. Total number of reads was 110142, of which 101266 reads (91.9%) included perfectly matching MID. The rest of the reads (8.1%) either included MID-tag with too many mismatches to be reliably identified or did not include a MID at all. In the latter case, the read usually appeared to be an artifact (for example primer-dimer), that is, not a real DNA sequence, which was confirmed by BLASTing these sequences against Genbank’s nucleotide database. After trimming away reads shorter than 100 bp we ended up with 41575 high quality reads. All control reactions proved to be free of contamination.

### Comparison of BLAST results from public and local databases

**Figure 2:**
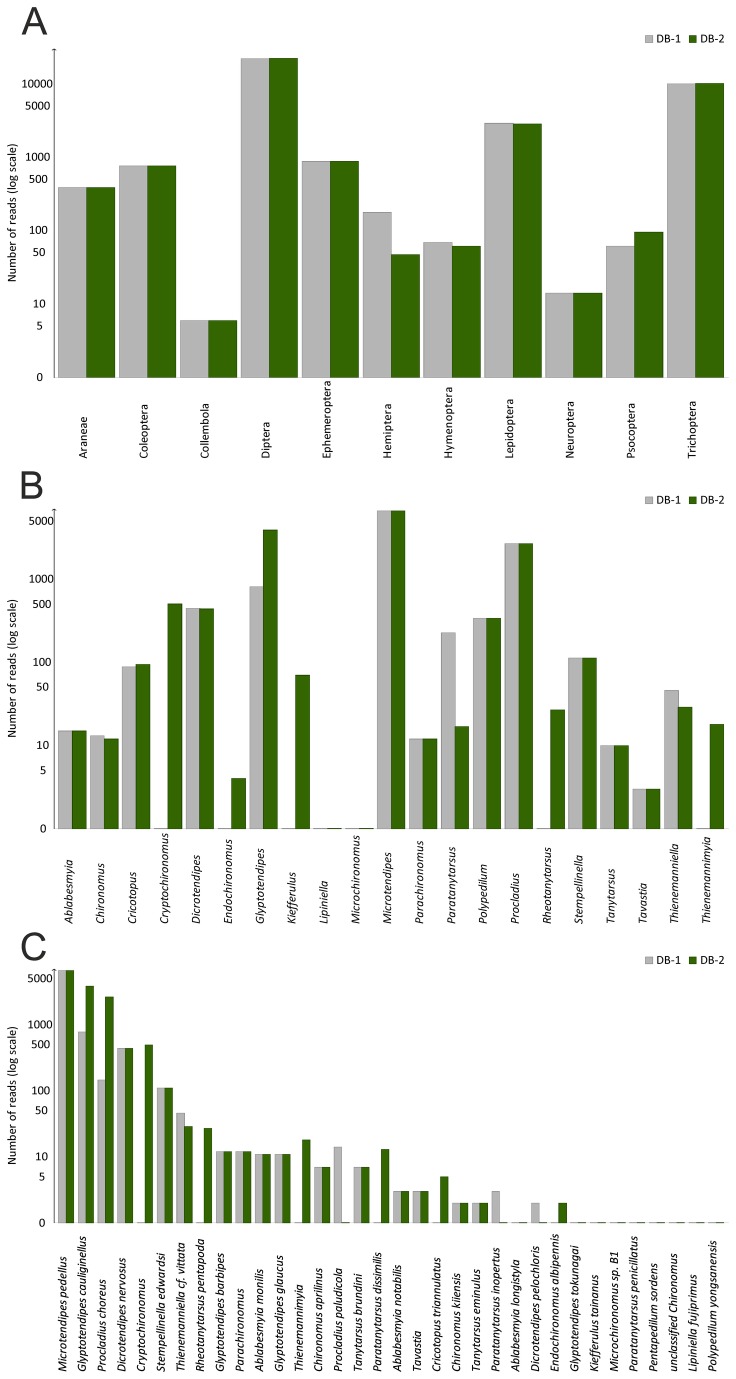
Comparison of results from public and private databases. The bar chart comparison of public databases (Genbank, BOLD) and public+private databases (Genbank, BOLD + FinBOL sequences) in identification of reads originating from Daubenton’s bats fecal samples: A. Comparing results at order level for all arthropods. B. Comparing results at genus level for family Chironomidae. C. Comparing results at species level for family Chironomidae.

We compared the blast results for the selected public databases and the local database first at order level. Most of the orders were equally represented in both blast outputs with Diptera being the most frequent order (Figure 2. A). For the order Hemiptera there were more hits in the public database whereas for the order Psocoptera there were more hits in the local database. We then compared genus and species level hits for the main prey group, the family Chironomidae of the order Diptera (Figure 2.B and C). Some of the genera and species are totally missing from the public database, or in some cases the sequences are available, but with incomplete identification information (Figure 2.B and C).

### BLAST results

The BLAST to the local database returned matches to 40769 (98%) of the query reads. Of these, 23253 query reads were qualified for further analysis, that is, at least 98% identity to database sequences and e-value < 1E-20. Altogether, 128 prey species from the Daubenton’s bats fecal DNA were identified, of which 79 with 100 % similarity to the reference database (Table S1). The effective number of species for the whole dataset was 99. The reads matched database sequences from three arthropod classes: most of the hits were assigned to class Insecta, and a small proportion to Arachnida and Collembola (Table 2). More than 70 % of all the species belonged to two proportionally most abundant orders: Diptera and Lepidoptera (Table 2). 

**Table 2:** Dietary taxa identified in Daubenton’s bats fecal samples at order level.

**Class**	**Order**	**Count**	**% prey taxa**	**% reads**
Arachnida	Araneae	3	2,3 %	1,0 %
Collembola	Entomobryomorpha	1	0,8 %	< 0,1 %
Insecta	Diptera	57	43,5 %	55,9 %
	Lepidoptera	40	30,5 %	7,4 %
	Trichoptera	11	8,4 %	29,7 %
	Hemiptera	7	5,3 %	0,5 %
	Neuroptera	5	3,8 %	0,2 %
	Coleoptera	3	2,3 %	2,3 %
	Ephemeroptera	2	1,5 %	2,8 %
	Psocoptera	2	1,5 %	0,2 %

Number of dietary taxa of all the arthropod orders found from the faeces and their proportion of all the dietary taxa (n = 131) and proportion of reads assigned to the taxon.

**Figure 3:**
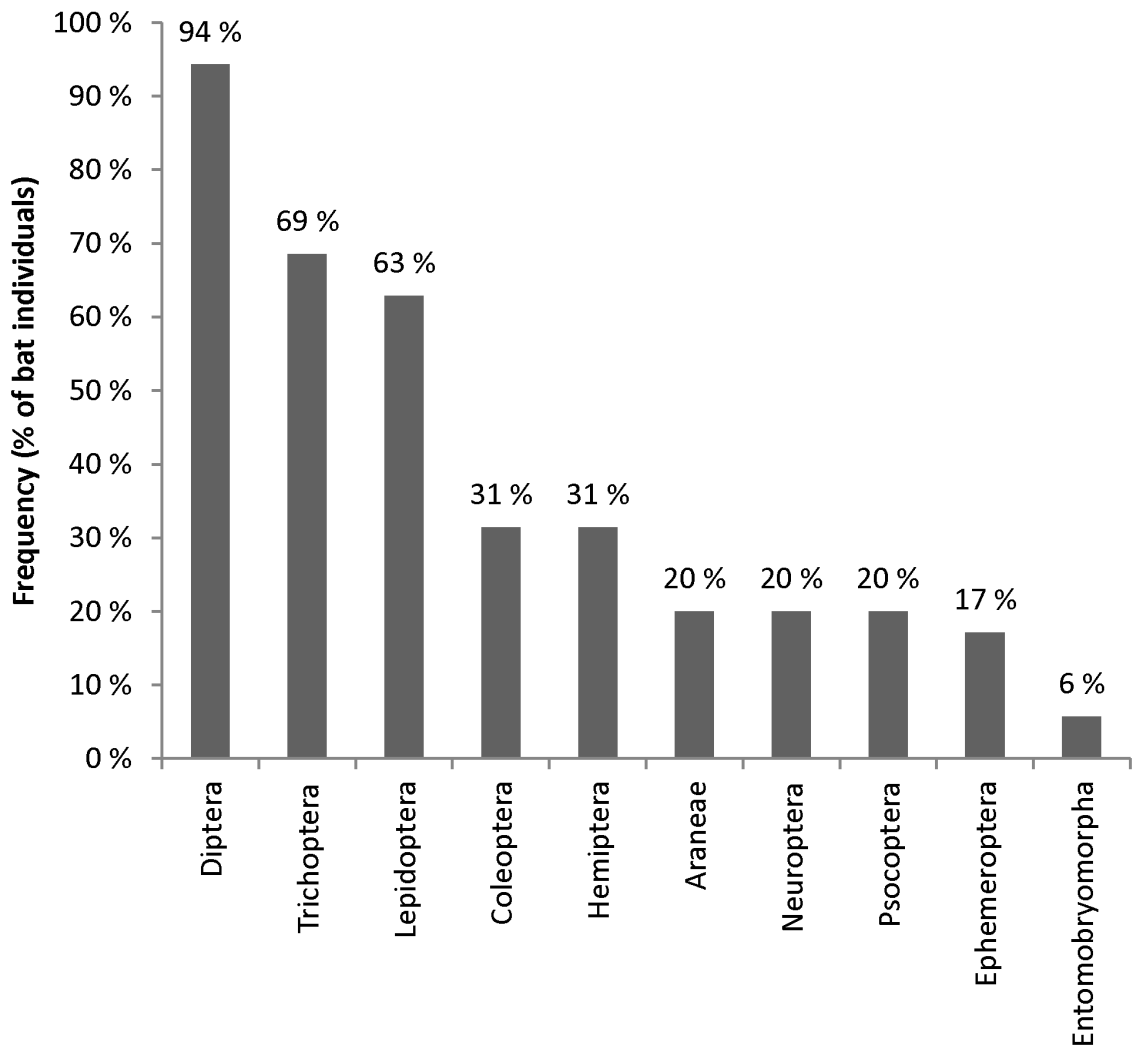
The most frequent arthropod orders in the Daubenton’s bats diet. The abundance of all the arthropod orders occurring in the Daubenton’s bats diet. The bar chart shows the frequency of the order in all the sampled bat individuals (n = 34).

**Figure 4:**
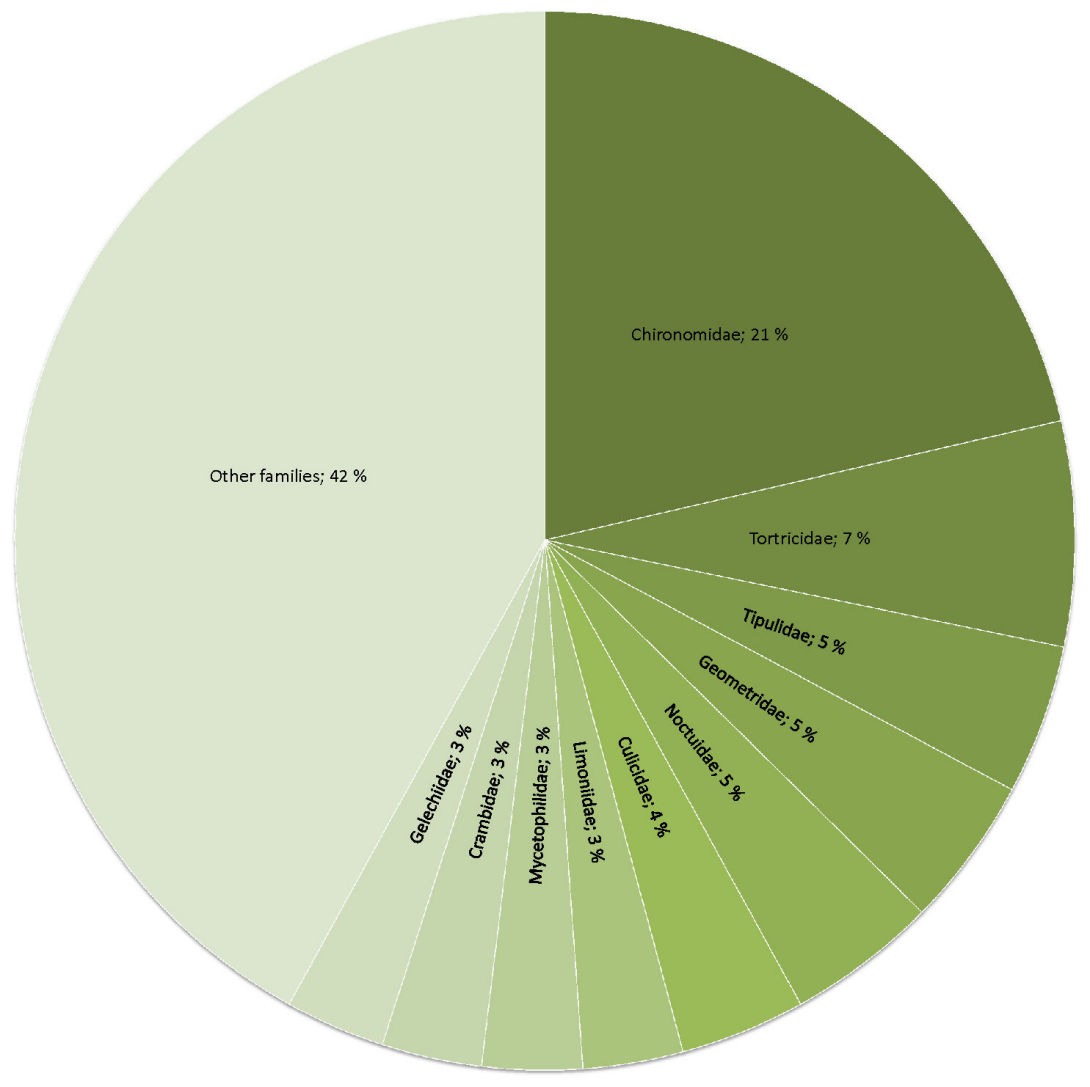
Most abundant families in the Daubenton’s bats diet. Pie chart shows proportions of the dietary families (n=49) obtained from Daubenton’s bat fecal DNA extractions amplified using arthropod-specific mtDNA .

The insect order Diptera was found in 94% of samples, Trichoptera 69%, and Lepidoptera 63% (Figure 3). More than every fifth (21 %) dietary species belonged to one single dipteran family Chironomidae (Figure 4), although there were a total of 49 families identified (Table S1). The most common prey family was the dipteran family Chironomidae, which was found in 89% of all the samples (Table 3). Other frequent families included the caddisfly family Leptoceridae, the moth family Tortricidae and another dipteran family, Tipulidae (Table 3). The most common prey species were non-biting midges (Diptera: Chironomidae) *Microtendipes pedellus* (50%), *Glyptotendipes cauliginellus* (44%), and *Procladius ferrugineus* (41%). The number of different prey species identified in each sample varied from two to twenty four. On average, one bat individual consumed 10 prey species.

**Table 3:** The most abundant (at least 20%) families in the Daubenton’s bats diet.

**Family**	**Abundance**	**Frequency**
Chironomidae	31	91 %
Leptoceridae	16	47 %
Tortricidae	14	41 %
Tipulidae	13	38 %
Limnephilidae	13	38 %
Phryganeidae	11	32 %
Limoniidae	10	29 %
Crambidae	10	29 %
Elateridae	9	26 %
Aphididae	9	26 %
Nycteribiidae	8	24 %
Muscidae	7	21 %
Gelechiidae	7	21 %

### Prey species diversity

**Figure 5:**
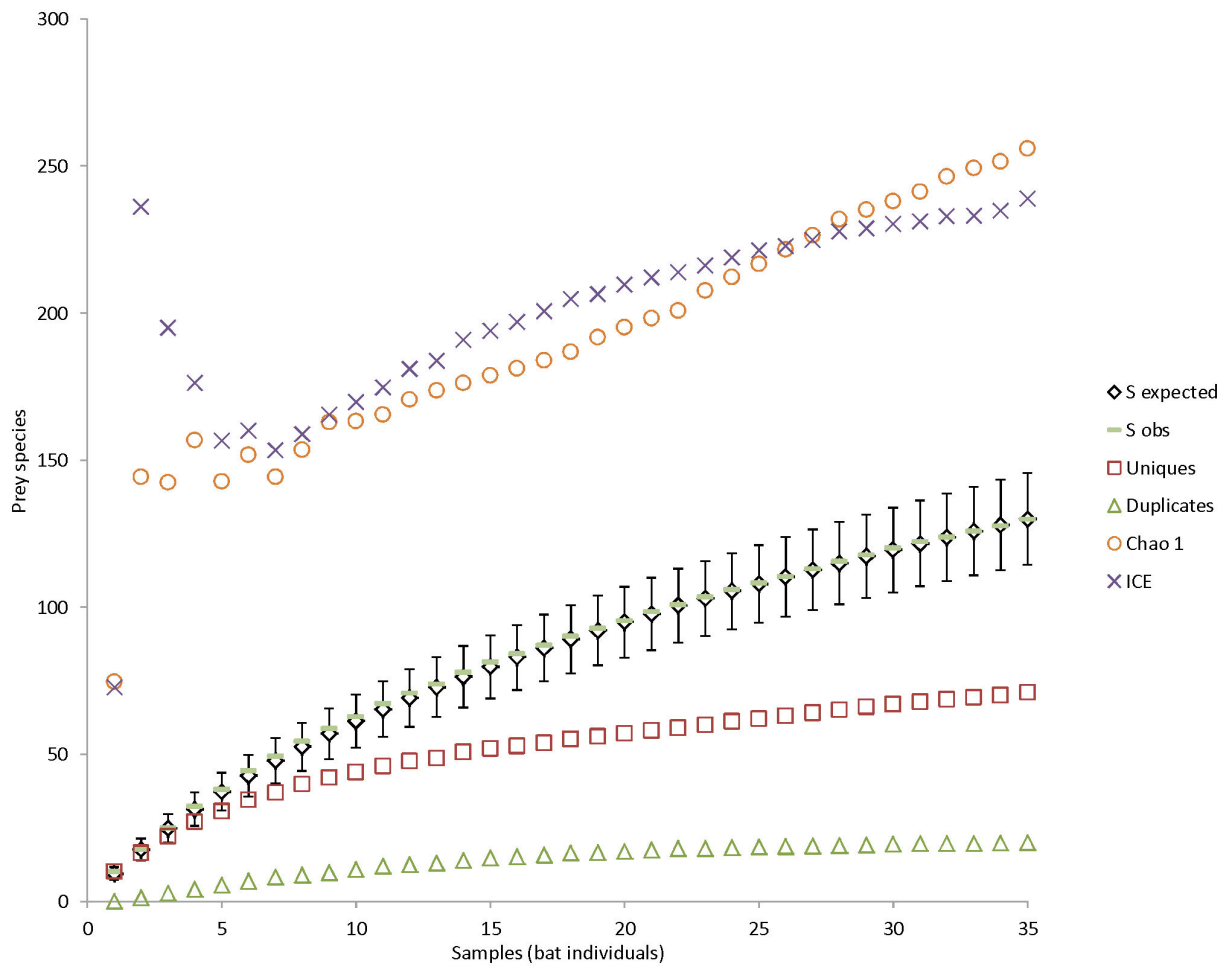
The prey species accumulation curve based on individual bat samples. The y-axis shows number of samples (*t*) accumulated. S_expected_, expected number of prey species in *t* pooled samples with 95% confidence intervals. S_obs_, observed number of prey species in *t* pooled samples. Uniques and duplicates, species observed only once or twice (respectively) in all the pooled samples. Chao1, incidence-based (presence/absence) non-parametric species richness estimator. ICE, Incidence coverage-based non-parametric species richness estimator. See text for references.

**Figure 6:**
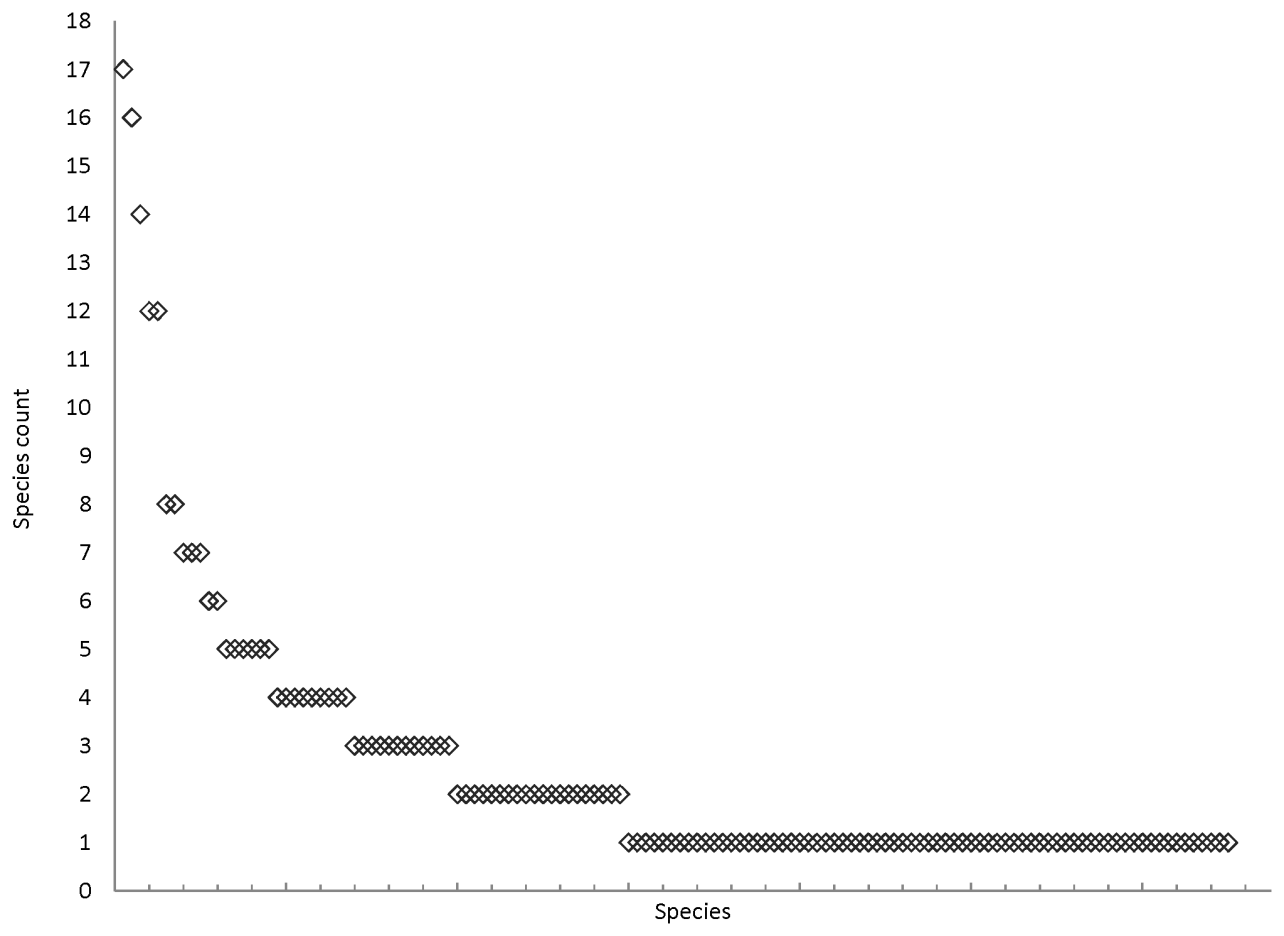
Dietary species’ dominance-diversity relationship. Log_10_ percentage of species occurrences are plotted against species rank (from most abundant to least abundant).

**Figure 7:**
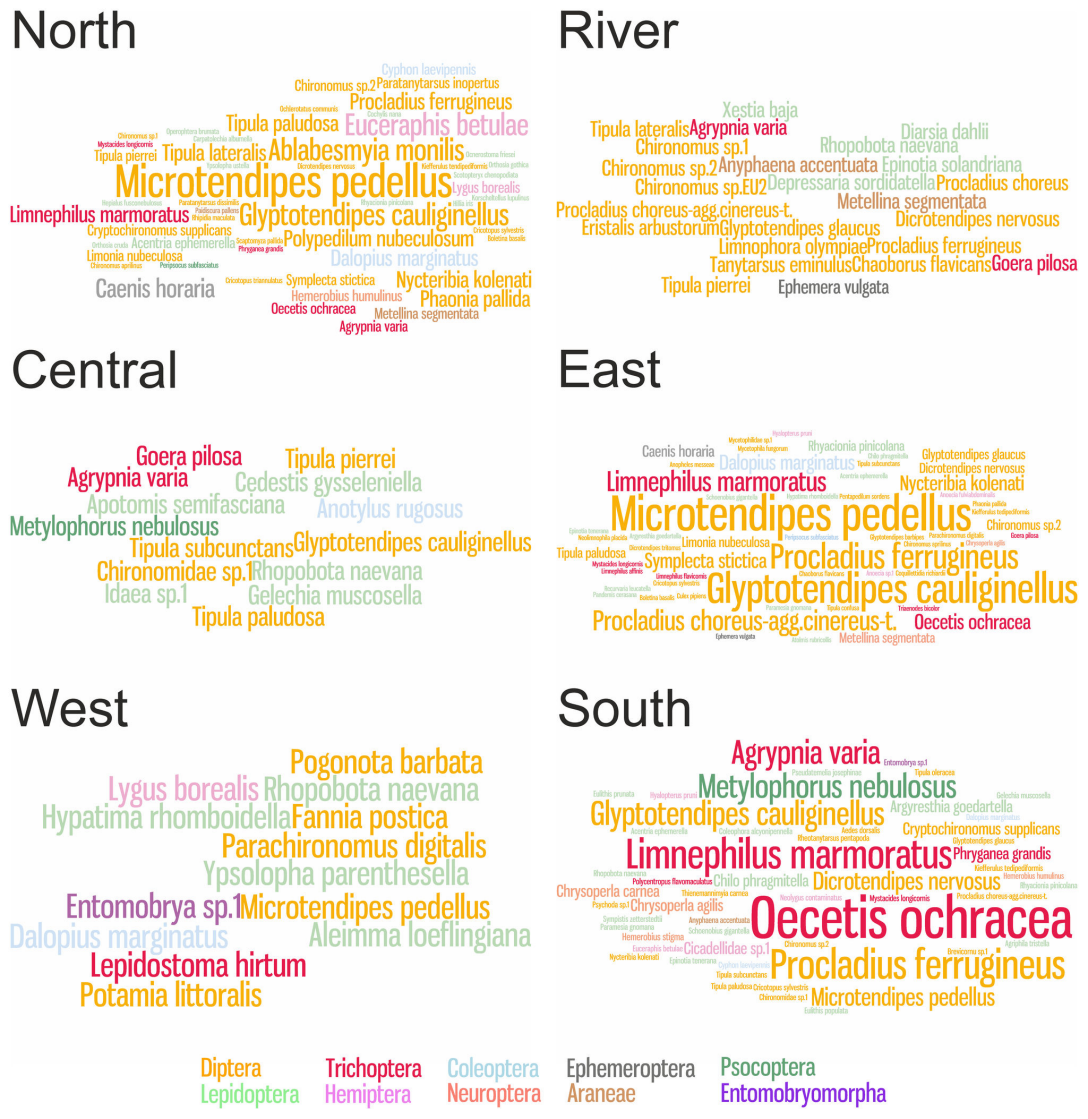
Daubenton’s bats prey species by frequency and taxonomic order. Species identification cloud of prey species identified from Daubenton’s bats fecal DNA samples on six sampling regions. Font size represents the proportional occurrence of each prey species in all the samples sequenced. See Figure 1 for regions on the map.

The species accumulation curve shows, that when resampled, every bat individual adds more species to the prey species list (Figure 5). The number of uniques (species occurring only once) and duplicates (species occurring only twice) are also high. Both non-parametric diversity estimators (Chao1 and ICE) predict overall species diversity higher than observed. The prey species rank abundance plot clearly indicates, that there are a few very dominant species in the diet, and many species occurring only rarely (Figure 6). The species identification cloud indicates that insects in the order Diptera are among the most consumed species in all regions (Figure 7). A trichopteran species, the mass-emerging *Oecetis ochraea*, is the most frequent single species only at a single location (South). The built-in comparison tool used for database comparison in MEGAN uses NCBI taxonomy (available at http://www.ncbi.nlm.nih.gov/taxonomy) as such, and for this reason our final prey species (Table S1) list differs from the chironomid genera and species list used in database comparison analysis (Figure 2). 

## Discussion

In this study we present the first accurate dietary species list for a widespread insectivorous predator, Daubenton’s bat (*Myotis daubentonii*) in Finland. With the help of the extensive local barcode library we were able to accurately and reliably identify 128 dietary species from the fecal samples.

### Molecular methods for dietary studies

We analysed 34 fecal pellets collected straight from bats while handling them. The library preparation protocol [38] was slightly modified in order to analyze fecal samples. The two sequencing runs did not produce as much data as we expected, but the data are nevertheless useful in demonstrating the method. Based on the preliminary results obtained in this study, we can conclude that Daubenton’s bats in the Archipelago Sea feed mainly on dipteran insects, especially of the family Chironomidae. The present conclusion is in agreement with previous studies on the diet of the Daubenton’s bat, in which almost all fecal pellets contained traces of chironomids [10,13,14,25]. The second most frequent prey groups are caddisflies and small moths, which were found to be equally frequent. This finding contradicts earlier studies, which seemed to find that moths are only marginal prey items for Daubenton’s bat, occurrences varying from very low to medium [26–28,30–32]. This may be due to variation in sampling habitats and different analysis methods, which vary from anecdotal evidence or direct observation of hunting bats [29], prey remain analysis [28], morphological analysis of fecal remains [10,13,14,26,27,30–32], and analysis of stomach contents [53,54]. It seems that while morphological identification of highly degraded remains yields similar results as DNA-based methods, the resolution of identification is not as good [3,12]. Based on the above we come to conclusion that modern sequencing technology, here Ion Torrent PGM, is suitable for accurate dietary studies.

### Importance of local DNA barcode library for molecular approach

In comparison to numerous earlier studies on insectivorous bat diets [3,9–14,17,25–33,53–56], the application of novel molecular techniques and an extensive DNA barcoding reference library in this study helped to truly identify dietary objects at the species level. Furthermore, with additions to the library, we can also apply the method to other bat species. As an improvement for future studies, more individuals should be sampled throughout the field season for temporal variation from several sites, and technical replicates such as several fecal pellets from a single individual and PCR replicates need to be analyzed.

### Daubenton’s bats dietary summary and taxonomic report of prey groups

Overall prey species richness for Daubenton’s bat was high: 128 identified species in 49 families and 10 arthropod orders. Although high, the number of prey species is in accordance with earlier studies on Daubenton’s bats diet [10,14,25]. All of the main prey species are aquatic or related to water habitat [57]. Many of the other prey species are also, for example many moths occurring only once or twice, using shore vegetation as a food source in their larval stage. The dietary composition of main prey groups was found to be similar across all three years, making the results more robust. Although the species richness of total diet was high, the effective number of species (true diversity) using species occurrence frequencies was lower (98) because of the few dominant species and many rare species. According to the resampling of the individual bat diets, the overall species richness is likely to be higher than observed, but there is little doubt that the observed main dietary groups are already revealed by this study. Overall, there were many arthropod groups and many species present in the diet. Here we will take an overview of taxonomic groups, and discuss the commonness and importance of various arthropod groups in Daubenton’s bats diet.

#### Class Arachnida

Three different spider species were found (*Anyphaena accentuate, Metellina segmentata*, and *Paidiscura pallens*). Two of these species are common medium-sized spiders living in the vegetation and trees (*A. accentuata and M. segmentata*). The third one (*P. pallens*), is a rare and very small species living in the oak forests in south-western Finland ([58]; S. Koponen, *pers. comm.*) and it was indeed found in the diet of a bat collected near an oak forest. *Metellina segmentata* was found from 5 bat individuals from three different localities. These spiders are non-aquatic and were perhaps caught when hanging from the web filament, straight from the vegetation or as “drop-outs” from the water surface. Although spiders are consistently found in the diet of Daubenton’s bats, they are usually at low frequencies [10,25] and thus cannot be considered to be important prey objects for Daubenton’s bat.

#### Class Collembola

Springtails (Collembola) were found from the diet of two young female bats, probably consumed at the roosting site, for example a bird-nesting box packed with moss. These springtails seem to be consumed by accident while grooming, and do not seem to be of importance for the bats’ nutrition.

#### Class Insecta

Three beetle species in three families (Elateridae: *Dalopius marginatus*, Scirtidae: *Cyphon laevipennis*, Staphylinidae: *Anotylus rugosus*) were found in the diet, consumed by all sex and age groups. *D. marginatus* and *A. rugosus* are very common and distributed over all of Finland, but *C. laevipennis* occurs only in southernmost Finland, however being very common there (Finnish Coleoptera Expert Groups web site at http://www.luomus.fi/elaintiede/kovakuoriaiset/index.htm; M. Pentinsaari, *pers. comm.*).

Most of the craneflies (Diptera: Nematocera) are common and tied to shores or wet habitat ([59]; J. Salmela, *pers. comm.*). Of the true flies, *Scaptomyza pallida, Pogonota barbata*, *Fannia postica*, and *Phaonia pallida* are common in all of Finland and *Potamia littoralis* occurs in some places at southern Finland (K. Winqvist, *pers. comm.*). One muscid fly species (*Limnophora olympiae*) has not been reported from Finland, but may be a true finding since it is occurring at least in Norway and Denmark (J. Kahanpää, *pers. comm.*). One of the dipteran “prey” species is a parasitic bat fly *Nycteribia kolenatii*, living in bat fur and sucking blood from the host animal. This parasitic species was likely consumed while the bat was cleaning its fur. All of the midges and other dipteran prey species (Diptera:Chironomidae, Chaoboridae) are common in the region ([60]; L. Paasivirta, *pers. comm.*). Chironomids are among the most common mass emerging insects in the area, and constitute the major part of the Daubenton’s bats diet.

The mayfly prey species (Ephemeroptera) are common and occurring in the area (J. Ilmonen, *pers. comm.*). The hemipteran species (aphids, leaf hoppers, and true bugs) are common in the study area (V. Rinne, *pers. comm.*). The prey moths found in the diet are common or quite common in southern Finland (A. Teräs, M. Mutanen, *pers. comm.*). The *Chrysoperla* species in order Neuroptera are common insects. The other species in this group *Hemerobius humulinus* and *H. stigma* (Neuroptera), are indeed common insects and are known to be predators of aphids [57].

The trichopteran species found in the diet of the bats are common and known to occur in the habitat and area ([61]; A. Rinne, J. Salokannel, *pers. comm.*). Some caddisflies are mass emerging, and it seems that these species are an important nutrition source for Daubenton’s bat.

Two species of bark lice (Psocoptera) are found in the diet (*Peripsocus subfasciatus* and *Metylophorus nebulosus*), both commonly occurring in the area (Finnish Hemiptera Expert Group psocopteran atlas available at http://www2.sci.utu.fi/projects/biologia/elainmuseo/hemi/psoc/psocmaps.htm). These insects are forest-dwelling species, eating lichen from tree surface [62]. In our data only male bats were found to consume insects in this group. It has been shown that males do fly further to eat [63], and this pattern might be the result of that kind of behavior. However, due to the small sample size, this question cannot be properly addressed. In any case, Psocoptera does not constitute a major prey group for the nutrition of Daubenton’s bats. 

The most common family in the whole dataset is the family Chironomidae. There are more than 700 chironomid species in Finland alone, making them the most species rich semi-aquatic insect group [60]. When comparing all the species found in the six sampling sites, chironomids constitute the majority of diet at all sites. One chironomid species (*Microtendipes pedellus*) was found in half of the sampled fecal pellets. However, it seems that while a major part of the diet of Daubenton’s bats consists of chironomids and caddisflies, they are versatile predators capable of catching various prey types in many ways. 

Yet there are some aquatic and terrestrial groups avoiding being eaten by bats. Perhaps the best known example are moths (Lepidoptera) with the ability to jam the bat echolocation [64–67], but also the very common aquatic insect group, the water-striders (Heteroptera: Gerridae), was absent from the diet. These insects are known to have a method to escape bats [68]. It may be that water striders in Finland have a similar adaptation and are able to escape the bats in to the dense common reed zone. Another very common aquatic group missing in the Daubenton’s bats diet are aquatic and semiaquatic beetles, which have been found to be consumed by bats elsewhere [69]. Either the Daubenton’s bat does not prefer aquatic beetles as a food item, or these beetles have indeed some yet unknown mechanism to avoid being caught. 

To summarize, based on our results, the question of Daubenton’s bats prey species is not fully answered, but we feel confident to conclude that in Finland it consumes mainly chironomids, caddisflies, and many species of moths. All of the prey species were common in the region and it appears that the bats are utilizing what is commonly available.

## Conclusions

The method described here can yield important information on the ecology and conservation of species, on part of the predator and prey. Our method is also readily applicable to many other insectivorous predators, such as birds. It also lends a possibility to recognize species in the faeces, which are too small or disintegrate in the guts preventing identification. Most likely the greatest advantage however is the fact that this method is non-invasive and samples can even be collected from day roosts without handling the animals. This is a welcome advantage of the method on part of the studied animal and the scientist dealing with the legislation of protected species in order to sample.

## Supporting Information

Table S1 **The species identified in the faeces of Daubenton’s bat diet in this study for each bat individual and sampling site.** The bottom row shows the number of prey species for each bat individual. Summary columns on the right show from how many bat individuals diet the prey species was found (Occurrences), the percentage frequency of each prey species (Frequency), and percentage match to reference database (Match to database).
(PDF)

Supporting information S1 **The information from the first Ion Torrent run carried out for this study.**
(PDF)

Supporting information S2 **The information from the second Ion Torrent run carried out for this study.**
(PDF
