# Supplementary material for: Next Generation Sequencing of Fecal DNA Reveals the Dietary Diversity of the Widespread Insectivorous Predator Daubenton’s Bat (Myotis daubentonii) in Southwestern Finland
Source: PLoS One. 2013 Nov 27;8(11):e82168. doi: 10.1371/journal.pone.0082168 (PMC3842304; doi:10.1371/journal.pone.0082168)
Supplement: Table S1 — The species identified in the faeces of Daubenton’s bat diet in this study for each bat individual and sampling site. The bottom row shows the number of prey species for each bat individual. Summary columns on the right show from how many bat individuals diet the prey species was found (Occurrences), the percentage frequency of each prey species (Frequency), and percentage match to reference database (Match to database). (PDF) [file pone.0082168.s001.pdf]

			Site / Band number	Brinkhall			Hemsundet						Korjaustelakka				Kukonpaa	Nautelankoski	Pakinainen	Rauhala							Seili				Tamminiemi									
Class	Order	Family	Species	1673	1790	2045	878	2062	2066	2079	2080	2082	1778	1973	2026	2053	2070	2099	1706	1829	2434	2435	2437	2438	2439	2479	2407	2409	2411	2413	868	889	1086	1373	1851	2015	2038	Occurrences *	Frequency (%) **	Match to database (%) ***
Arachnida																																								
	Araneae	Anyphaenidae	*Anyphaena accentuata*															1											1									2	5,9	100,0
		Tetragnathidae	*Metellina segmentata*															1					1		1								1			1		5	14,7	100,0
		Theridiidae	*Paidiscura pallens*																															1				1	2,9	100,0
Collembola																																								
	Entomobryomorpha	Entomobryidae	*Entomobrya sp.1*																1											1								2	5,9	100,0
Insecta																																								
	Coleoptera	Elateridae	*Dalopius marginatus*		1														1				1		1					1		1	1	1				8	23,5	100,0
		Scirtidae	*Cyphon laevipennis*																								1					1					1	3	8,8	100,0
		Staphylinidae	*Anotylus rugosus*														1																					1	2,9	100,0
	Diptera	Chaoboridae	*Chaoborus flavicans*		1													1																				2	5,9	100,0
		Chironomidae	*Ablabesmyia monilis*																													1	1	1		1		4	11,8	100,0
			Chironomidae sp.1								1						1																					2	5,9	98,5
			Chironomus aprilinus																	1													1					2	5,9	100,0
			Chironomus sp.1												1			1																				2	5,9	99,2
			Chironomus sp.2	1														1			1								1					1		1		6	17,6	99,4
			Chironomus sp.EU2															1																				1	2,9	99,1
			Cricotopus sylvestris							1					1					1																		3	8,8	100,0
			Cricotopus triannulatus																															1				1	2,9	100,0
			Cryptochironomus supplicans								1	1			1																		1					4	11,8	100,0
			Dicrotendipes nervosus		1		1						1					1			1						1		1									7	20,6	100,0
			Dicrotendipes tritomus																						1													1	2,9	98,5
			Glyptotendipes barbipes																					1														1	2,9	98,5
			Glyptotendipes cauliginellus					1			1	1		1	1		1			1	1	1	1	1		1	1					1	1					15	44,1	100,0
			Glyptotendipes glaucus		1													1							1					1								4	11,8	98,2
			Kiefferulus tendipediformis				1								1								1															3	8,8	100,0
			Microtendipes pedellus	1	1	1	1	1					1						1	1	1		1	1			1				1	1	1	1		1		17	50,0	100,0
			Parachironomus digitalis																1				1															2	5,9	99,1
			Paratanytarsus dissimilis																													1						1	2,9	98,7
			Paratanytarsus inopertus																													1				1		2	5,9	100,0
			Pentapedilum sordens		1																																	1	2,9	100,0
			Polypedilum nubeculosum																												1	1	1					3	8,8	100,0
			Procladius choreus															1																				1	2,9	98,2
			Procladius choreus-agg.cinereus-t.															1		1	1	1				1			1									6	17,6	100,0
			Procladius ferrugineus					1			1			1	1			1		1	1	1		1	1		1		1	1			1					14	41,2	100,0
			Rheotanytarsus pentapoda																											1								1	2,9	98,4
			Tanytarsus eminulus															1																				1	2,9	99,4
			Thienemannimyia carnea																										1									1	2,9	99,2
		Culicidae	*Aedes dorsalis*																										1									1	2,9	100,0
			Anopheles messeae																				1															1	2,9	99,4
			Coquillettidia richiardii																				1															1	2,9	98,0
			Culex pipiens																						1													1	2,9	99,2
			Ochlerotatus communis																												1							1	2,9	99,0
		Drosophilidae	*Scaptomyza pallida*										1																									1	2,9	100,0
		Fanniidae	*Fannia postica*																1																			1	2,9	99,0
		Limoniidae	*Limonia nubeculosa*												1						1		1														1	4	11,8	100,0
			Neolimnophila placida																						1													1	2,9	99,4
			Rhipidia maculata																																	1		1	2,9	100,0
			Symplecta stictica											1						1	1			1													1	5	14,7	100,0
		Muscidae	*Limnophora olympiae*															1																				1	2,9	98,7
			Phaonia pallida											1	1					1																		3	8,8	100,0
			Potamia littoralis																1																			1	2,9	100,0
		Mycetophilidae	*Boletina basalis*																		1													1				2	5,9	100,0
			Brevicornu sp.1				1																															1	2,9	100,0
			Mycetophila fungorum																				1															1	2,9	99,4
			Mycetophilidae sp.1																						1													1	2,9	98,1
		Nycteribiidae	*Nycteribia kolenati*												1	1				1	1		1					1									1	7	20,6	100,0
		Psychodidae	*Psychoda sp.1*				1																															1	2,9	99,4
		Scathophagidae	*Pogonota barbata*																1																			1	2,9	98,2
		Syrphidae	*Eristalis arbustorum*															1																				1	2,9	98,1
		Tipulidae	*Tipula confusa*																				1															1	2,9	100,0
			Tipula lateralis												1			1															1				1	4	11,8	99,1
			Tipula oleracea																									1										1	2,9	98,6
			Tipula paludosa										1		1		1					1				1		1				1						7	20,6	100,0
			Tipula pierrei														1	1																1			1	4	11,8	99,1
			Tipula subcunctans														1					1						1										3	8,8	100,0
	Ephemeroptera	Caenidae	*Caenis horaria*		1	1								1	1																					1		5	14,7	100,0
		Ephemeridae	*Ephemera vulgata*			1												1																				2	5,9	100,0
	Hemiptera	Anoeciidae	*Anoecia fulviabdominalis*																							1												1	2,9	100,0
		Aphididae	*Anoecia sp.1*																							1												1	2,9	98,5
			Euceraphis betulae						1						1																1	1	1					5	14,7	100,0
			Hyalopterus pruni																							1			1									2	5,9	100,0
		Cicadellidae	*Cicadellidae sp.1*				1																				1											2	5,9	99,4
		Miridae	*Lygus borealis*												1				1														1					3	8,8	100,0
			Neolygus contaminatus				1																															1	2,9	100,0
	Lepidoptera	Argyresthiidae	*Argyresthia goedartella*	1			1																						1									3	8,8	100,0
		Coleophoridae	*Coleophora alcyonipennella*				1																															1	2,9	100,0
		Crambidae	*Acentria ephemerella*																							1			1				1			1		4	11,8	100,0
			Agriphila tristella																										1									1	2,9	100,0
			Chilo phragmitella				1		1													1																3	8,8	100,0
			Schoenobius gigantella						1														1															2	5,9	100,0
		Elachistidae	*Depressaria sordidatella*															1																				1	2,9	98,2
		Erebidae	*Atolmis rubricollis*																				1															1	2,9	100,0
		Gelechiidae	*Carpatolechia alburnella*																													1						1	2,9	100,0
			Gelechia muscosella					1									1																					2	5,9	100,0
			Hypatima rhomboidella																1					1														2	5,9	100,0
			Recurvaria leucatella	1																																		1	2,9	98,7
		Geometridae	*Eulithis populata*						1																													1	2,9	98,4
			Eulithis prunata						1																													1	2,9	100,0
			Idaea sp.1														1																					1	2,9	99,0
			Operophtera brumata																													1						1	2,9	100,0
			Scotopteryx chenopodiata												1																							1	2,9	100,0
		Hepialidae	*Hepialus fusconebulosus*												1																							1	2,9	100,0
			Korscheltellus lupulinus												1																							1	2,9	100,0
		Lypusidae	*Pseudatemelia josephinae*				1																															1	2,9	100,0
		Noctuidae	*Diarsia dahlii*															1																				1	2,9	98,2
			Hillia iris																												1							1	2,9	98,1
			Orthosia cruda																												1							1	2,9	99,1
			Orthosia gothica																												1							1	2,9	99,1
			Sympistis zetterstedtii						1																													1	2,9	99,1
			Xestia baja															1																				1	2,9	99,2
		Tortricidae	*Aleimma loeflingiana*																1																			1	2,9	100,0
			Apotomis semifasciana														1																					1	2,9	100,0
			Cochylis nana																														1					1	2,9	99,4
			Epinotia solandriana															1																				1	2,9	100,0
			Epinotia tenerana					1																1														2	5,9	99,2
			Pandemis cerasana																						1													1	2,9	100,0
			Paramesia gnomana	1			1																															2	5,9	99,1
			Rhopobota naevana							1							1	1	1																			4	11,8	100,0
			Rhyacionia pinicolana																				1	1				1									1	4	11,8	100,0
		Yponomeutidae	*Cedestis gysseleniella*														1																					1	2,9	98,7
			Ocnerostoma friesei																													1						1	2,9	100,0
		Ypsolophidae	*Ypsolopha parenthesella*																1																			1	2,9	100,0
			Ypsolopha ustella																												1							1	2,9	99,1
	Neuroptera	Chrysopidae	*Chrysoperla agilis*																			1						1	1									3	8,8	100,0
			Chrysoperla carnea																								1		1									2	5,9	99,4
		Hemerobiidae	*Hemerobius humulinus*				1							1																	1							3	8,8	100,0
			Hemerobius stigma																								1											1	2,9	100,0
	Psocoptera	Peripsocidae	*Peripsocus subfasciatus*											1							1																	2	5,9	99,4
		Psocidae	*Metylophorus nebulosus*				1	1			1						1										1											5	14,7	100,0
	Trichoptera	Goeridae	*Goera pilosa*			1											1	1																				3	8,8	100,0
		Lepidostomatidae	*Lepidostoma hirtum*																1																			1	2,9	100,0
		Leptoceridae	*Mystacides longicornis*												1					1									1									3	8,8	100,0
			Oecetis ochracea						1	1	1												1	1	1		1	1	1	1		1				1		12	35,3	100,0
			Triaenodes bicolor			1																																1	2,9	98,4
		Limnephilidae	*Limnephilus affinis*																				1															1	2,9	100,0
			Limnephilus flavicornis																					1														1	2,9	98,0
			Limnephilus marmoratus								1	1				1				1			1	1	1		1	1	1				1		1			12	35,3	100,0
		Phryganeidae	*Agrypnia varia*				1	1									1	1									1		1						1	1		8	23,5	100,0
			Phryganea grandis				1				1																						1					3	8,8	100,0
		Polycentropodidae	*Polycentropus flavomaculatus*																										1									1	2,9	100,0
			Number of prey species per bat individual:	5	7	5	16	7	7	3	8	3	4	7	17	2	14	24	13	11	11	7	18	11	11	7	13	8	18	6	9	14	16	8	2	10	7			

* This is the number of bat individuals to have the prey species in their diet

** Percentage of bat individuals to have this species on their diet

*** The highest percentage of reads to match the prey species the reference database
