# Supplementary material for: Next Generation Sequencing of Fecal DNA Reveals the Dietary Diversity of the Widespread Insectivorous Predator Daubenton’s Bat (Myotis daubentonii) in Southwestern Finland
Source: PLoS One. 2013 Nov 27;8(11):e82168. doi: 10.1371/journal.pone.0082168 (PMC3842304; doi:10.1371/journal.pone.0082168)
Supplement: Supporting information S1 — The information from the first Ion Torrent run carried out for this study. (PDF) [file pone.0082168.s002.pdf]

Run Summary

3M
Total Bases

15
Key Signal

23%
Bead Loading

ISP Density

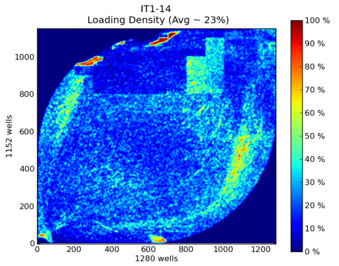

39,233
Total Reads

24%
Usable Sequence

ISP Summary

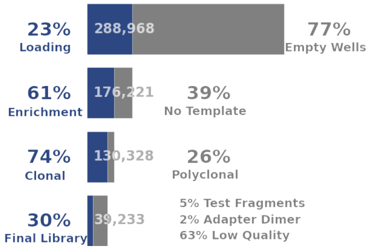

77 bp
Mean Read Length

Read Length

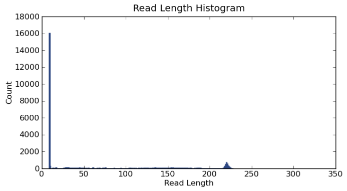

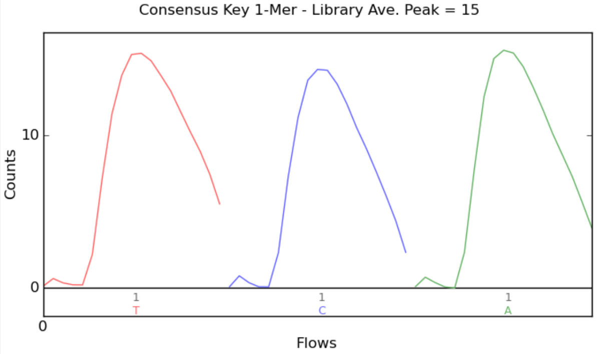

**Addressable Wells**	**1,262,555**	
With ISPs	288,968	22.9%
Live	176,221	61.0%
Test Fragment	10,411	05.9%
Library	165,810	94.1%

**Library ISPs**	**165,810**
Filtered: Polyclonal	45,893
Filtered: Low Quality	78,381
Filtered: Primer Dimer	2,303
**Final Library ISPs**	**39,233**

Test Fragment	Reads	Percent 50AQ17	Read Length Histogram
**TF_D**	**3,221**	**87%**	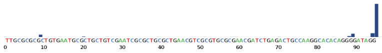
**TF_A**	**3,089**	**85%**	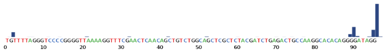

ion torrent
by life technologies

Alignment Summary *(aligned to)*

None	
Total Alignment Bases	Average Coverage Depth of Reference

alignment_rate_plot.png

%
Mean Raw Accuracy 1x

base_error_plot.png

	AQ17	AQ20	Perfect
**Total Number of Bases [Mbp]**	None	None	None
**Mean Length [bp]**			
**Longest Alignment [bp]**			
**Mean Coverage Depth**			

Filtered_Alignments_Q10.png

Filtered_Alignments_Q17.png

Filtered_Alignments_Q20.png

Filtered_Alignments_Q47.png

Analysis Details

**Run Name**	R_2012_01_27_20_06_24_user_IT1-14
**Run Date**	Jan. 28, 2012, 3:06 a.m.
**Run Flows**	440
**Projects**	pcr-eero
**Sample**	15indies
**Library**	none
**PGM**	IT1
**Flow Order**	TACGTACGTCTGAGCATCGATCGATGTACAGC
**Library Key**	TCAG
**TF Key**	ATCG
**Chip Check**	Passed
**Chip Type**	314R
**Chip Data**	single
**Barcode Set**	
**Analysis Name**	Eero-PCR-Jan2012
**Analysis Date**	March 3, 2013, 10:31 p.m.
**Analysis Flows**	440
**runID**	63S90

Software Version

**Torrent_Suite**	3.4.1
**host**	ph1
**ion-alignment**	3.4.3-1
**ion-analysis**	3.4.7-1
**ion-dbreports**	3.4.26-1
**ion-gpu**	3.0.0-1
**ion-pipeline**	3.4.16-1
**ion-plugins**	3.4.19-1
**ion-torrentr**	3.4.5-1
**Script**	17.1.0
**LiveView**	300
**DataCollect**	196
