# Supplementary material for: Next Generation Sequencing of Fecal DNA Reveals the Dietary Diversity of the Widespread Insectivorous Predator Daubenton’s Bat (Myotis daubentonii) in Southwestern Finland
Source: PLoS One. 2013 Nov 27;8(11):e82168. doi: 10.1371/journal.pone.0082168 (PMC3842304; doi:10.1371/journal.pone.0082168)
Supplement: Supporting information S2 — The information from the second Ion Torrent run carried out for this study. (PDF [file pone.0082168.s003.pdf]

Run Summary

8.9M
Total Bases

43
Key Signal

23%
Bead Loading

ISP Density

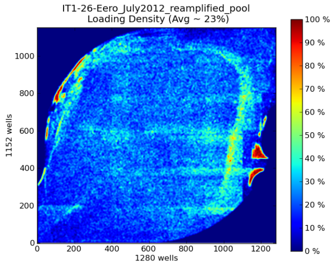

70,909
Total Reads

46%
Usable Sequence

ISP Summary

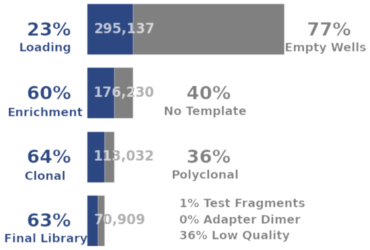

126 bp
Mean Read Length

Read Length

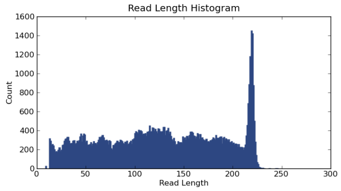

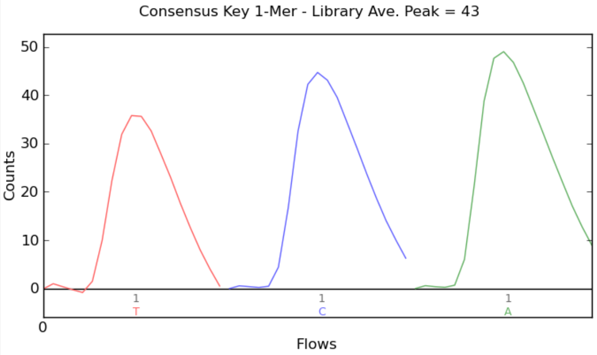

**Addressable Wells**	**1,262,576**	
With ISPs	295,137	23.4%
Live	176,230	59.7%
Test Fragment	21,504	12.2%
Library	154,726	87.8%

**Library ISPs**	**154,726**
Filtered: Polyclonal	63,198
Filtered: Low Quality	20,591
Filtered: Primer Dimer	28
**Final Library ISPs**	**70,909**

Notes: Earlier run produced only a few reads. This time the pooled library is re-amplified before template preparation -EJW

Alignment Summary *(aligned to)*

None	
Total Alignment Bases	Average Coverage Depth of Reference

alignment_rate_plot.png

%
Mean Raw Accuracy 1x

base_error_plot.png

	AQ17	AQ20	Perfect
**Total Number of Bases [Mbp]**	None	None	None
**Mean Length [bp]**			
**Longest Alignment [bp]**			
**Mean Coverage Depth**			

Filtered_Alignments_Q10.png

Filtered_Alignments_Q17.png

Filtered_Alignments_Q20.png

Filtered_Alignments_Q47.png

ion torrent
by life technologies™

Analysis Details

**Run Name**	R_2012_06_28_05_03_03_user_IT1-26-Eero_July2012_reamplified_pool
**Run Date**	June 28, 2012, 12:03 p.m.
**Run Flows**	520
**Projects**	NSG-BATFP
**Sample**	Re-AmpJuly2012
**Library**	none
**PGM**	IT1
**Flow Order**	TACGTACGTCTGAGCATCGATCGATGTACAGC
**Library Key**	TCAG
**TF Key**	ATCG
**Chip Check**	Passed
**Chip Type**	314R
**Chip Data**	single
**Barcode Set**	
**Analysis Name**	Eero-PCR-July2012-re3
**Analysis Date**	March 4, 2013, 1:45 a.m.
**Analysis Flows**	520
**runID**	7XY1U

Software Version

**Torrent_Suite**	3.4.1
**host**	ph1
**ion-alignment**	3.4.3-1
**ion-analysis**	3.4.7-1
**ion-dbreports**	3.4.26-1
**ion-gpu**	3.0.0-1
**ion-pipeline**	3.4.16-1
**ion-plugins**	3.4.19-1
**ion-torrentr**	3.4.5-1
**Script**	18.1.6
**LiveView**	345
**DataCollect**	210
**OS**	19
**Graphics**	18
